# CD8+ T cell infiltration in breast and colon cancer: A histologic and statistical analysis

**DOI:** 10.1371/journal.pone.0190158

**Published:** 2018-01-10

**Authors:** James Ziai, Houston N. Gilbert, Oded Foreman, Jeffrey Eastham-Anderson, Felix Chu, Mahrukh Huseni, Jeong M. Kim

**Affiliations:** 1 Department of Pathology, Genentech, Inc., South San Francisco, California, United States of America; 2 Department of Biostatistics, Genentech, Inc., South San Francisco, California, United States of America; 3 Department of Oncology Biomarker Development, Genentech, Inc., South San Francisco, California, United States of America; 4 Department of Cancer Immunology, Genentech, Inc., South San Francisco, California, United States of America; University of Pécs Medical School, HUNGARY

## Abstract

The prevalence of cytotoxic tumor infiltrating lymphocytes (TILs) has demonstrated prognostic value in multiple tumor types. In particular, CD8 counts (in combination with CD3 and CD45RO) have been shown to be superior to traditional UICC staging in colon cancer patients and higher total CD8 counts have been associated with better survival in breast cancer patients. However, immune infiltrate heterogeneity can lead to potentially significant misrepresentations of marker prevalence in routine histologic sections. We examined step sections of breast and colorectal cancer samples for CD8+ T cell prevalence by standard chromogenic immunohistochemistry to determine marker variability and inform practice of T cell biomarker assessment in formalin-fixed, paraffin-embedded (FFPE) tissue samples. Stained sections were digitally imaged and CD8+ lymphocytes within defined regions of interest (ROI) including the tumor and surrounding stroma were enumerated. Statistical analyses of CD8+ cell count variability using a linear model/ANOVA framework between patients as well as between levels within a patient sample were performed. Our results show that CD8+ T-cell distribution is highly homogeneous within a standard tissue sample in both colorectal and breast carcinomas. As such, cytotoxic T cell prevalence by immunohistochemistry on a single level or even from a subsample of biopsy fragments taken from that level can be considered representative of cytotoxic T cell infiltration for the entire tumor section within the block. These findings support the technical validity of biomarker strategies relying on CD8 immunohistochemistry.

## Introduction

Breast and colorectal cancers are the second and third most common malignancies worldwide, respectively. Between 2008 and 2015, breast cancer incidence has increased by more than 20%, while mortality has increased by 14% and it now represents one in four of all cancers in women [1]. Colorectal cancer, at the same time, has decreased 3.2% per year in incidence although mortality has remained stable [2]. Although new chemotherapeutic regimens have improved overall and disease-free survival in breast and colorectal cancer patients over the past decade, the benefits in overall survival are heterogeneous and the outlook remains suboptimal, particularly in colorectal cancer.

Disease progression in cancer patients is determined not only by the histologic and molecular features of the tumor but also by the host response, particularly the immune response. Observations that inflammatory cell density is higher in tumor tissue than in non-adjacent normal tissue in breast cancer patients [3] and that histologic distributions of CD4 and CD8 T cell subsets in, for instance liver metastases of colorectal cancer, can be correlated with clinical stage and outcome in colorectal cancer patients [4] emphasize that there is a topography to tumor control by the immune system. As such, much attention has been given to characterizing the immune contexture of tumors and determining the relationship of immune populations within the tumor microenvironment to clinical behavior, prognosis and therapeutic response. While associations between prevalence and outcome for some immune populations, such as B cells, remain unclear, the positive prognostic value of cytotoxic (CD8) tumor infiltrating lymphocytes (TIL) has been demonstrated in multiple solid tumor types including breast [5,6] and colorectal cancer [7,8,9] as well as melanoma [10,11,12,13], bladder [14,15], prostate [16,17], ovarian [18,19], pancreatic [20,21], and, when associated with proliferative index, renal cell carcinoma [22]. Studies in colorectal cancer have shown CD8+ and CD45RO+ cell prevalence and distribution to be superior to traditional UICC clinical staging in predicting colorectal cancer patient outcomes [8].

In addition to the prognostic impact of CD8 levels across multiple cancer indications, densities of intratumoral CD8+ T cells are associated with response to anti-PD-1 (pembrolizumab) treatment in melanoma and mismatch repair deficient cancers, including colon cancer [23,24]. Therefore, enumerating CD8+ T cell densities from archival tumor samples may serve as a predictive biomarker for melanoma and colon cancer patients who benefit from PD-1: PD-L1 blockade. Since CD8+ T cells are hypothesized to be the final effector cells that mediate tumor cell killing, changes in intratumoral CD8+ T cell dynamics serve as a pharmacodynamic marker of clinical activity in certain indications. Together, intratumoral CD8 densities are employed as a predictive and pharmacodynamic biomarker in melanoma and colon cancer, and a prognostic marker in multiple indications.

Although most studies show improved prognosis with increased tumor-associated cytotoxic T cells, some reports have demonstrated conflicting results. For non-small cell lung cancer, several studies associated increased CD8 with improved patient outcomes [25,26,27], but others showed no effect on survival [28], or decreased amounts CD8+ infiltrates have been correlated with improved survival [29]. In addition, CD8+ T cell infiltration and its association with survival in a renal cell carcinoma patients has not demonstrated significance [30,31]. While the heterogeneity of these findings is partly attributable to the variety of methods and endpoints assessed, the heterogeneity of immune infiltration in the tumor microenvironment is also potentially confounding. Examining the heterogeneity of PD-L1 staining and variability in interpretation, Rehman et al. have demonstrated that the variability in PD-L1 signal within a block is greater than between blocks from the same tumor and that one block is sufficient to represent the heterogeneity for a tumor sample [32]. As such, it is possible that significant misrepresentations of lymphocyte populations can occur in single routine histologic sections due to uneven distribution of immune cells throughout the tumor tissue within one block. This issue can be further complicated in evaluation of smaller tumor biopsies compared to full-section slides, where representation of the tumor and its immune contexture is even more limited.

When a biomarker such as CD8 is employed in a clinical setting, the effects of assay variability, whether attributable to analytical characteristics of the assay itself or due to pre-analytic sample properties such as sample preparation and sample type (e.g., resection, slide, or core), could potentially have broader implications for clinical trial study design, study conduct, and the interpretation of results at the end of trial [33,34,35,36,37]. In practice, clinical trials, particularly those in earlier stages of drug development, are rarely powered to distinguish prognostic from predictive effects of a biomarker [35,37,38], and using an underperforming assay may only exacerbate this endeavor. In the case of an assay that dichotomizes a patient population into distinct subgroups of “diagnostic-negative” and “diagnostic-positive,” an assay with relatively reduced ability to accurately identify patients in a diagnostic-positive subpopulation may result in longer clinical trial recruitment and readout timelines due to a need for enhanced screening to enroll a sufficient number of diagnostic-positive patients. Inclusion of diagnostic-positive patients who benefit from a targeted therapy into the analysis of a diagnostic-negative subgroup may in some cases lead to an inflated estimate of treatment benefit in a patient subgroup where no treatment effect exists, thereby altering the overall perceived benefit-risk ratio of the therapy under evaluation. Finally, and perhaps most important for practitioners sizing clinical trials, differential misclassification of diagnostic-negative patients can dilute the treatment effect of a predictive biomarker in a diagnostic-positive subgroup, resulting in a net loss of power as more patients will be needed to detect the same amount of clinical benefit around which the trial was originally designed [39,40]. Power considerations are made even more difficult in the setting of a continuous biomarker or in any setting where the assay cut-off may be unknown [36,39,40].

In light of these practical considerations: what constitutes an adequate histologic evaluation of immune contexture, particularly in characterizing cytotoxic T cell infiltration of a tumor for prognosis, predicting response to immunotherapy, and for monitoring on-treatment pharmacodynamics? To inform practice of histologic T cell biomarker interpretation, we examined step sections of breast and colorectal cancer samples for CD8 T cell prevalence by immunohistochemistry, and performed *in silico* biopsy sampling of these sections to determine CD8 variability within a tumor block and between simulated biopsy samples. Numbers of CD8+ T cells were obtained by digital image analysis for each level and simulated biopsy. CD8 counts between levels of a tissue sample were fit to a linear variance model to determine the statistical significance of the CD8 count variability. Similarly, in simulated core biopsies, the degree of variability in CD8 counts was assessed and related to the overall ‘true’ block count to determine the number of cores necessary for adequate sampling. Our results show that CD8+ T cell numbers are highly homogenous throughout colon and breast cancer tissue section and that a single virtual core biopsy can potentially adequately represent tumor-associated cytotoxic T cell prevalence, though estimates improve with increasing sample number.

## Materials and methods

### Case selection and sectioning

Formalin-fixed paraffin-embedded (FFPE) breast cancer and colon cancer samples were procured. Breast cancer samples were procured from Avaden Biosciences and included both ductal (8) and medullary (4) carcinoma cases. Cases contained between 20% and 80% tumor in the regions analyzed by visual estimate of area. Patient ages for breast samples ranged from 40–82 years and AJCC stages I-IV were represented. Colorectal cancer samples were procured from Conversant Bio and included moderately differentiated adenocarcinoma (9), mucinous carcinoma (1) and rectal (2) carcinoma cases. Samples contained between 20% and 60% tumor in the regions analyzed by visual estimate of area. Patient ages ranged from 43–89 years, and stages I and II disease were represented.

**Fig 1:**
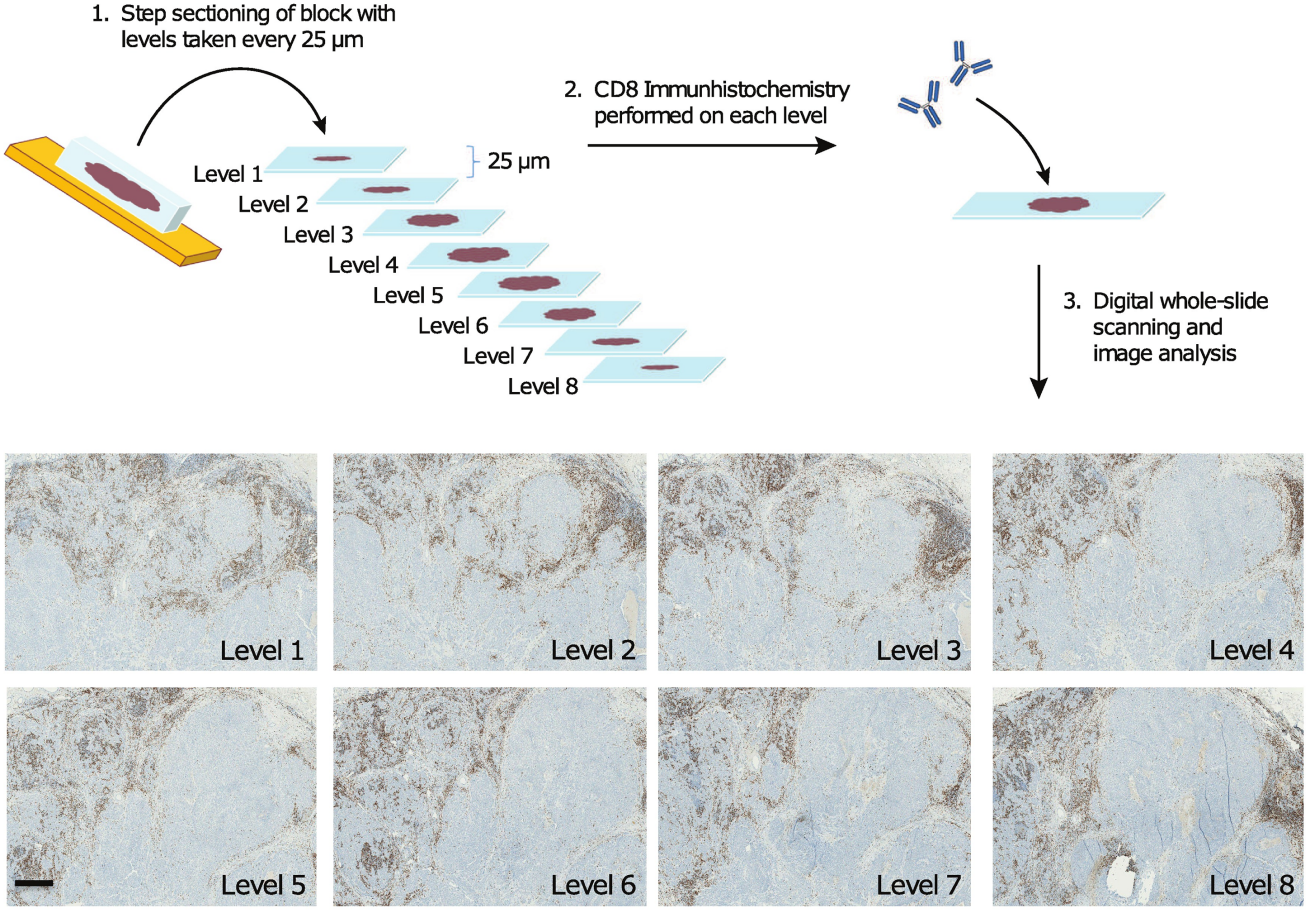
Sample workflow for immunohistochemistry and image analysis. Tumor blocks were sectioned and levels for CD8 immunohistochemistry taken at 25 μm intervals. Stained slides were scanned and the tumor area and immediately adjacent stroma was manually designated by a pathologist on all slides. All nucleated cells as well as CD8+ cells within the defined area were identified and counted by image analysis. Simulated core biopsies were identified by creating a grid of rectangular regions over the entire image, each approx. 2mm^2^. in size. Rectangular regions that overlapped with at least 0.7 mm^2^ of manually identified region were analyzed. Scale bar illustrated in “Level 1” panel equals 500 μm.

Tissue blocks were serially sectioned and four serial levels were taken approximately every 10 μm until the block was exhausted or tissue significantly reduced yielding eight sets of 4 serial sections per case available for staining. For any given block, the second level of each set of serial sections was stained for CD8, leading to an assessment of CD8 prevalence every 25 μm (Fig 1).

### Immunohistochemical staining

Immunohistochemistry (IHC) was performed on 4 μm thick formalin-fixed, paraffin-embedded tissue sections mounted on glass slides. All IHC steps were carried out on the Ventana Discovery XT automated platform (Ventana Medical Systems; Tucson, AZ). Sections were treated with Cell Conditioner 1, standard time, and then incubated in primary antibody, CD8 (C8-144B, Dako, cat. # M7103) at a working concentration of 0.157 mg/ml for 60 minutes at room temperature. Specifically bound primary antibody was detected by the OmniMap anti-rabbit HRP detection kit, followed by ChromoMap DAB (Ventana Medical Systems; Tucson, AZ). The sections were counterstained with Hematoxylin II (Ventana Medical Systems; Tucson, AZ), dehydrated, and cover-slipped. Positive staining controls were performed in parallel using human tonsil and negative controls were performed using IgG1 isotype antibody.

### Digital imaging and analysis

Whole slide images were acquired with a Nanozoomer 2.0-HT automated slide scanning platform (Hamamatsu, Hamamatsu City, Shizuoka Pref., Japan) at 200x final magnification. Scanned slides were analyzed in the Matlab software package (version R2012b by Mathworks, Natick, MA) as 24-bit RGB images. Tumor regions were manually identified at a macroscopic level. Simulated core biopsies were identified by creating a grid of rectangular regions over the entire image, each approx. 2 mm^2^ in size. Rectangular regions that overlapped with at least 0.7 mm^2^ of manually identified region were analyzed. Individual cells were identified using an algorithm based on radial symmetry [41]. Each cell was then scored as positive or negative for DAB staining using a blue-normalized algorithm to identify brown pixels [42].

### Statistical analyses

All statistical analyses were conducted in the R environment for statistical computing [43]. In addition to computing summary statistics (means, medians, ranges), CD8 staining data were also analyzed using a linear model framework both pooled across tissue types and within each tissue type individually. The data were first analyzed using a fixed effects linear model (ANOVA) with patient and section both treated as factors. In this manner, the contribution of handling section as a factor in the model could be assessed relative to the more parsimonious submodel in which section was not considered. The percent of variability in the model explained by the patient and section effects was estimated. As a second pass, variance component analysis was continued using a linear mixed effects model where a patient-level random effect shared by measurements (sections) taken on the same subject (tumor sample or block) was incorporated into a model for CD8+ percent staining (random intercept model). The intraclass correlation coefficient (ICC), bounded by 0 and 1, and estimated as the ratio of the variance of the patient random effect to the total variability in the model, was calculated as a summary measure for the similarity of sections taken from a given tumor sample.

Resampling methods were employed to answer questions about the utility of using a smaller number of sections for determining CD8+ status. The mean CD8-positive percent staining value across sections within a tumor sample was treated as the true expression value for that specimen, and the median value across all tumor samples was selected a candidate cut-off for CD8+ staining. Resampling was performed 1000 times, (i) by resampling smaller numbers of sections—e.g., one or two from the original 8—with replacement within the original, fixed set of 25 tumor samples to assess the effects of within-tumor heterogeneity, and (ii) by resampling with replacement first tumors and then sections within tumors to better assess the effects of biological and technical variability in determining CD8+ status in future samples of patient tumor blocks.

A total of 198 slides sectioned from the 25 blocks were further subdivided into simulated smaller biopsy fragments ("cores") of maximum size ~2 mm^2^, each with a minimum tumor area of at least 33% (median number of simulated biopsy fragments per slide: 57, range: 2–161). A key question was how many simulated smaller biopsies would be needed to adequately approximate the CD8 IHC staining and assay performance of (i) the entire slide and (ii) the entire tumor block (pooled across slides). Sampling 1000 times without replacement, the mean CD8 percent positive staining was estimated using increasing numbers of cores (1–5 cores) and by taking either the mean or maximum of the subsampled biopsy fragments as an estimate summary measure. To allow for less-biased resampling without replacement of up to 5 cores, analysis (i) was restricted to 183 slides with at least 15 identified fragments (analysis-restricted median number of simulated biopsy fragments per slide = 59, range: 15–161, S1 and S2 Figs). The difference between the mean (of maximum) of the core biopsies selected at each round of resampling and the mean (or maximum) of the cores *not* selected (the “out-of-bag” sample) was calculated as a summary measure. Local regression (loess) fits were also obtained in order to characterize the variability associated with sample fragments as a function of mean CD8 percent staining [44]. Assay performance was also evaluated in terms of accuracy, sensitivity and specificity of the summary measure estimates relative to the slide or block mean intensity (i.e., “truth”) across a variety of potential candidate cut-offs for CD8-positive percent staining, e.g., 1%, 2%, 5% and 10% positivity.

## Results

### Prevalence and distribution of CD8+ T cell infiltrates in breast and colon cancer cases

In order to determine whether sampling error contributes to variation in intratumoral T cell counts, we sectioned through and enumerated CD8+ T cells in 13 primary colorectal adenocarcinoma and 12 breast carcinoma (6 ductal adenocarcinoma, 6 medullary carcinoma) cases (1 FFPE block per case). Step sections of breast and colorectal cancer samples were taken at approximately 25 μm intervals from FFPE samples and stained for CD8 using DAB-based chromogenic immunohistochemistry (Fig 1). Image analysis was performed on manually identified tumor regions, which included tumor margins. Samples were manually evaluated for adequacy and no significant background staining or labeling of non-lymphocyte cell populations were noted on any CD8 immunohistochemistry slide. CD8+ cells were identified and calculated as a percentage of total cells identified by hematoxylin counterstain.

From the 12 breast cancer cases, 3 were noted to have significant tissue loss on deeper levels. Specifically, deepest sections (level 8) from two cases showed less than 40% of tissue remaining compared to the initial level. A separate case showed approximately 30% of tissue compared to the initial level on levels 7 and 8. Only one colon cancer sample showed significant tissue loss on one of eight levels. Since these changes could potentially introduce artifactual variability, statistical analyses that both included and excluded these levels were run.

Breast cancer samples showed a wide range of cytotoxic T cell infiltration varying from less than 1% (0.41%, ductal adenocarcinoma) to greater than 50% (55.9%, medullary carcinoma) marker-positive lymphocytes. Histology showed diffuse T-cell infiltration with most breast cancer cases showing CD8+ cells in comparable amounts at the tumor edge and center. Medullary carcinoma cases showed characteristically high lymphocyte infiltration and high CD8+ area (mean 24.0%, median 19.7%, pooled intrapatient SD: 1.7%), compared to ductal adenocarcinoma (mean 8.8%, median 5.9%, pooled intrapatient SD: 1.2%).

Colorectal cases showed, in general, somewhat less cytotoxic T cell infiltration than breast cancer cases (mean 3.8%, median 2.9%, intrapatient SD: 0.4%). The majority of CRC cases (12/13) showed less than 10% CD8+ T cells on any level in contrast to breast cancer samples where 50% (6/12) of cases showed less than 10% CD8+ prevalence. The maximum amount of CD8+ T cells was 13.6%. In contrast to breast cancer tissues, CD8+ T cells in colon cancer cases were confined largely the stroma with few infiltrating lymphocytes among epithelial cells.

### CD8 IHC assay performance characteristics

**Fig 2:**
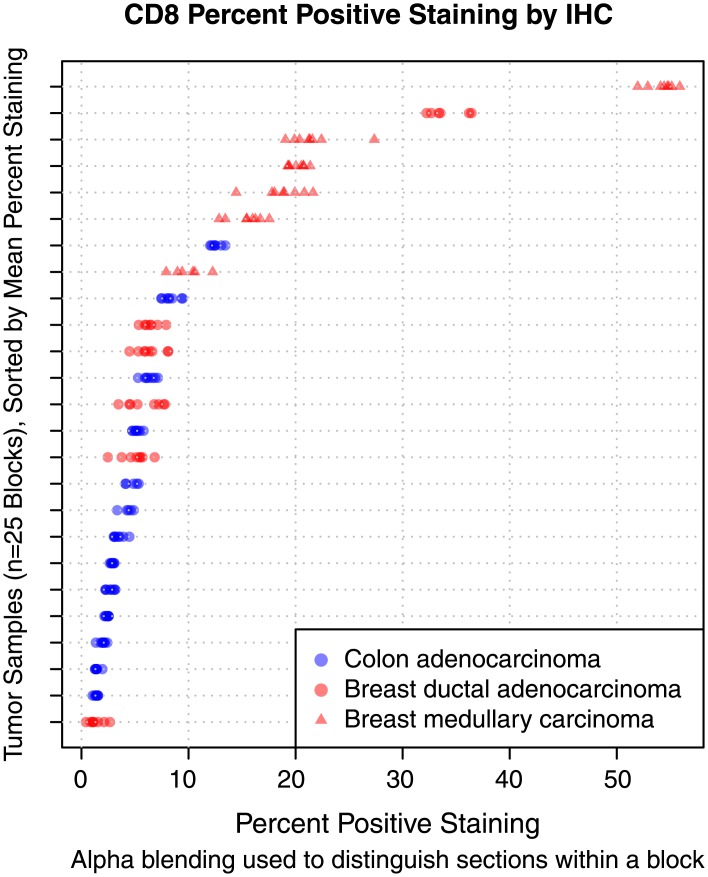
CD8 IHC repeated measures staining results for 25 breast (red) and colorectal (blue) carcinoma samples. Each tumor block was sectioned 8 times or until the sample was exhausted.

Assessment of full-face sections showed greater variability in CD8+ T cell prevalence between cases than between levels from an individual case. This observation also held when cases were divided by histologic type, such that cytotoxic T cell prevalence on serial sections varied more greatly between cases of ductal adenocarcinoma than within a case of ductal adenocarcinoma (Fig 2). However, minimal variation in cytotoxic T cell counts between levels within any given tumor block was observed over the entire observed dynamic range of CD8 staining (Fig 2). In a pooled analysis of all colon and breast samples, a linear model was fit using tumor block and slide section as explanatory variables for percent staining. Patient variability at the tumor block level accounted for 99.2% of the (biological) variability in the model. Even when the two most extreme tumor samples containing CD8+ expression > 30% were removed from the analysis (Fig 2), the patient-level effect still explained 95.8% of the total variability in the model. The effect of section variability failed to reach statistical significance in any analyses subgroup explored. The ICC calculated for percent CD8+ cells between sections within a tumor sample in the linear mixed effects model variance component analysis was 0.99. In order to characterize the variability within tumor type, separate models were also fit stratified by tumor type, and for the breast samples, within histologic subtype. From this analysis, percent variability (%) in CD8 counts between step sections from colonic adenocarcinoma (0.2%), medullary breast carcinoma (0.1%) and ductal adenocarcinoma (0.1%) blocks were minor. Together, these results suggest that variation in CD8 counts between cases for breast or colon samples is driven largely by interpatient biological effects and not due to inadequate sampling.

To determine whether a single slide is sufficient to assess the status of CD8+ T cell abundance, we next assessed the performance of this assay benchmarked against a median cut-off. In the resampling-based analysis, pooling breast and colon samples, a median (50th percentile) cutoff of 5.9% percent CD8+ staining was calculated for determining a patient’s CD8+ status. With a patient’s average CD8 expression computed across all sections being considered "truth,” patients were classified as CD8-high or CD8-low depending on whether or not their mean expression was above or below the median cut-off determined by the larger sample. Relative to the CD8 status determined by the mean CD8 expression, classifying patient samples by taking even just one slide at random resulted in 94.8% (95% CI: 88.0% -100%) positive agreement (concordance), with 94.5% simulated sensitivity in detecting “CD8-high” patients and 95.1% simulated specificity in detecting “CD8-low” patients (S3 Fig). Similar results were obtained when resampling both patients and sections within patients [95.2% positive agreement (95%CI: 84.0%-100%); 94.9% sensitivity and 95.5% specificity] (S3 Fig). No appreciable differences in assay performance were observed when significant tissue loss was excluded versus when they were included (data not shown). Therefore, for classifying tumor blocks using CD8 counts, the usage of an individual slide is not predicted to lead to misclassification due to insufficient tumor block sampling.

**Fig 3:**
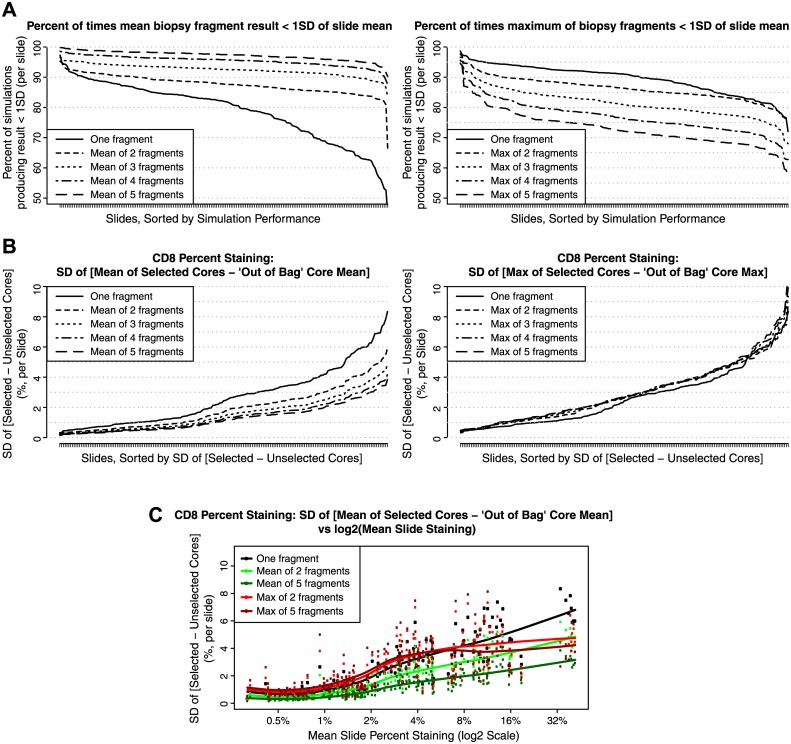
Slide-level biopsy simulation results. (A) Percent of times over 1000 rounds of simulations that the values obtained from sampling increased numbers of biopsy fragments produced a result within 1SD of the mean CD8 staining for a given slide. Calculating the mean over increased numbers of biopsies led to better estimates of the mean, while calculating maxima over the sample biopsies led to overestimates of a slide’s CD8 levels. Samples are sorted by increasing performance in terms of being able to produce an estimate within 1SD of total CD8 staining for that slide. (B) Estimates of the standard deviation of the difference between the mean or maximum of selected core biopsies and the mean or maximum of the out-of-bag or unselected cores on a given slide. Increased sampling leads to improvements in variability for when using means but not when using order statistics. (C) Estimates of the standard deviation of the difference between the mean or maximum of selected core biopsies and the mean or maximum of the out-of-bag or unselected cores on a given slide as a function of the mean CD8 percent positive staining for a slide. Loess fits are used to highlight mean performance over the observed dynamic range on both the log_2_ and observed percent staining scales. Increased sampling leads to improvements in variability when using means but not when using order statistics.

**Fig 4:**
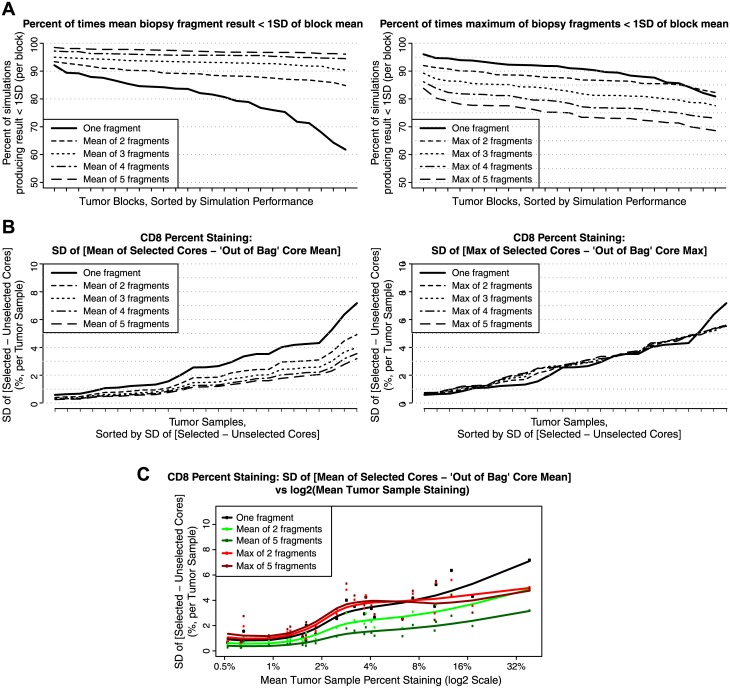
Block-level biopsy simulation results. (A) Percent of times over 1000 rounds of simulations that the values obtained from sampling increased numbers of biopsy fragments produced a result within 1SD of the mean CD8 staining for an entire tumor block. Calculating the mean over increased numbers of fragments led to better estimates of the mean, while calculating maxima over the sample biopsies led to overestimates of a sample’s CD8 levels. Samples are sorted by increasing performance in terms of being able to produce an estimate within 1SD of total CD8 staining for that tumor block. (B) Estimates of the standard deviation of the difference between the mean or maximum of selected core biopsies and the mean or maximum of the out-of-bag or unselected cores for a given tumor sample. Increased sampling leads to improvements in variability for when using means but not when using order statistics. (C) Estimates of the standard deviation of the difference between the mean or maximum of selected core biopsies and the mean or maximum of the out-of-bag or unselected cores for a given tumor sample as a function of the mean CD8 percent positive staining for that block. Loess fits are used to highlight mean performance over the observed dynamic range on both the log_2_ and observed percent staining scales. Increased sampling leads to improvements in variability when using means but not when using order statistics.

Compared to archival tumor blocks utilized for prognosis and predictive diagnostic studies, needle biopsies acquired for post-treatment pharmacodynamics analysis represent a smaller sample of the entire tumor, and may be prone to technical variation (S1 and S2 Figs). In practice, most investigators and patients are willing to provide a limited number of core biopsies, and not all of those core biopsies may be designated to measure the same biomarker. In order to assess the cost, as measured by variability, associated with using a core biopsy sample instead of a tumor cross-section, we performed virtual biopsy sampling from within slides and compared biopsy-based CD8+ cell prevalence to slide- and block-based measurements of staining. Rectangular fields of view of between 0.5 and 2.0 mm^2^ were digitally generated within manually selected tumor regions and the percentage of CD8+ cells were calculated for each field. Surprisingly, sampling just 1–5 biopsy fragments from an individual slide, and across slides, recapitulated CD8 staining observed both at the slide and tumor block level, respectively (Figs 3 and 4). Using the set of cores for a slide as a proxy for parent data with known mean and variance, sampling just one biopsy led to an estimate of CD8 staining that was within 1 standard deviation (SD) of the total slide staining in greater than 70% of simulations for 141 of the 183 slides analyzed (77.0%) and in over 80% of simulations for 100 slides (Fig 3A). Taking the mean of two biopsies produced estimates of CD8 staining that were within 1SD of the slide staining intensity in 182 slides (99.4%) in over 80% of simulations, with 42 (23.0%) of slides landing within the 1 SD range in over 90% of simulations (Fig 3A). Incremental performance gains were observed with more intensive sampling, with gains after sampling three cores becoming less pronounced (Fig 3A and 3C).

To mitigate against bias when sampling a subset of cores from a finite set of fragments, we estimated the differences in means of the fragments selected at each round of sampling with the means of the fragments not selected (the “out-of-bag” sample), and were thus able to approximate the standard deviations associated with sampling the subsets of cores relative to the rest of the tumor sample. Differences in means were centered around zero (unbiased), with decreases in variability again observed with increased sampling (Fig 3B) and incremental gains in performance observed after sampling three cores. The standard deviations were also plotted as a function of mean CD8 staining for the full set of cores and fitted with loess smoothers to highlight that the improvements in performance were observed over the full dynamic range of CD staining (Fig 3C). Similar trends were detected when using the same numbers of fragments to estimate CD8 staining pooled over all slides for each of the 25 blocks (Fig 4A and 4C).

While it may not be surprising that with increased sampling, the mean of an increasing number of core biopsies will approach the value which was measured on its parent slide or in an entire block with “square root *n*”-like convergence, some researchers may alternatively use the maximum percent staining value obtained on from a small number of biopsies as a summary measure. Our results demonstrate that the use of order statistics such as maxima should be carefully considered or avoided altogether if one is trying to get a representative assessment of overall staining within a tumor or individual. Using the maximum over increasing numbers of biopsies resulted in biased overestimates of CD8 staining relative to the mean CD8 positivity and in estimates which rapidly diverged from the mean +/- 1SD range of CD8 staining of the slide or block (Figs 3A and 4A). In addition to producing biased estimates, the use of the maximum did not result in improvements in variability relative to the out-of-bag mean or maximum of the unselected cores (Figs 3B, 3C, 4B and 4C)

**Fig 5:**
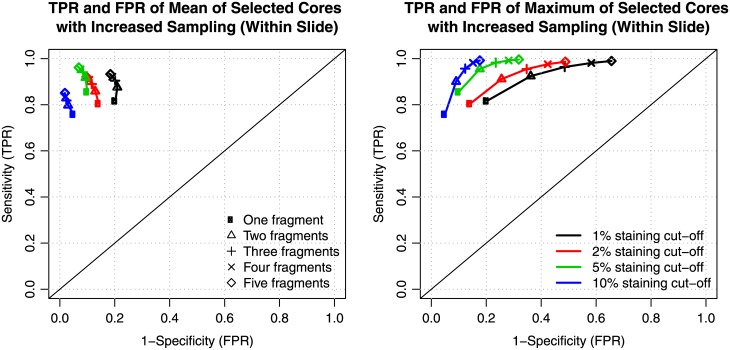
Simulation receiver-operator characteristic (ROC) curves. With increased sampling, there is increased performance of the CD8 IHC assay to classify as positive or negative for a given cut-off (different colors) when using the means of different numbers of sampled biopsies (left) and decreased performance when using maxima (right). Over the set of cut-offs (1%, 2%, 5%, 10%), the analysis treated the mean CD8 staining as the true intensity against which the results of the subsamples of biopsy fragments were evaluated. Similar results were obtained when benchmarking the staining result from core biopsies against the mean staining of the entire block (data not shown).

In addition to characterizing the bias and variability over the range of CD8 staining in our sample, receiver-operator characteristic (ROC) curves were used to display assay performance of the CD8 staining estimates obtained from the biopsies over a range of potential candidate staining cut-offs that could be considered in a clinical trial setting (1%, 2%, 5% and 10%). Here, the mean CD8 staining of a slide or block was used as the gold standard or "truth" against which CD8 positivity or negativity as determined by a set of simulated core biopsies was evaluated for a given cut-off. In terms of being able to correctly classify patients as being CD8-positive or CD8-negative, a high level of sensitivity and specificity was observed when comparing results from a single biopsy fragment to a sample’s status based on evaluation of the whole slide or block. For example, using a 5% cut-off, relative to the result obtained from a slide, the value for the biopsy fragment displayed on average 85.6% sensitivity and 89.9% specificity over 1000 rounds of simulation (Fig 5). Taking the mean of two biopsy fragments increased the sensitivity to 91.7% while maintaining specificity at 90.7% (Fig 5). Over the set of candidate cut-offs examined, as evidenced by movement towards the upper left corners of the ROC plots, increased sampling of cores led to increases in sensitivity and specificity of the biopsies’ ability to classify slides as CD8-positive or CD8-negative when using the mean of the biopsies as a summary measure (Fig 5). As expected, taking the maximum of the biopsies resulted in an overestimation of the sample’s mean CD8 staining, leading to higher occurrences of false positives (Fig 5). Together, our data suggest that although increased number of core biopsies resulted in enhanced CD8 assay sensitivity a single core biopsy is sufficient to represent CD8 levels, regardless of whether median or candidate cutoff is employed.

## Discussion

Predictive diagnostics for cancer immunotherapies have focused on the tumor-immune microenvironment and its composition. As such, histologic assessment of tumors and their immune contexture is becoming central to prognosticating clinical behavior in patients with solid tumors both by determining the amount of immune infiltrate and its composition, as well as prevalence of actionable immunotherapy targets, e.g. PD-L1, and prognostically relevant immune cell subtypes, including CD8+ T cells. However, variable sampling and interpretation methods including scoring algorithms and inter-pathologist variability can potentially confound histology as a prognostic method. As well, interpretation of more limited samples, such as core biopsies, raises questions about sampling adequacy and error. However, the extent and potential effect(s) of sample type and spatial heterogeneity of tumor immune infiltrates has not been detailed. The clinical and scientific importance of TIL assessment in breast cancer has been underscored by recent efforts to standardize histologic interpretation of TILs in patient samples, but it has been noted that TIL heterogeneity and adequacy of core biopsy samples versus tissue sections has not been formally characterized [45].

CD8+ cytotoxic T cells in particular have received attention not only because of their known role as cytolytic agents and demonstrated reactivity to tumor-derived self-epitopes [46] but also their prevalence and apparent positive prognostic effect in breast cancer as well as other tumor types. In particular, the presence of CD8+ cells in the tumor prior to the onset of neoadjuvant chemotherapy has been shown to predict response to therapy including pathological complete response particularly in triple-negative and HER2-expressing breast cancers [47,48,49,50]. In colon cancer, immunohistochemical assessment of CD3+ T cells and CD8+ T cells in the tumor microenvironment has been shown to more accurately predict patient outcome than traditional UICC staging and its utility as an adjunct to traditional UICC staging has been proposed [51]. Additionally, in colon cancer patients with microsatellite instability, CD8+ T cell density predicts response to anti-PD-1 treatment [24]. Given these findings and the lack of published evidence documenting the extent of TIL heterogeneity in whole tumor sections or biopsies, we evaluated CD8+ T cell prevalence in step sections from breast and colon tumor blocks and simulated core biopsy samples to determine the variability of tumor-associated CD8+ T cell prevalence between levels within a block and between simulated core biopsies and whole tissue sections as well as block means.

Our results demonstrate that the prevalence of CD8+ T cells is highly consistent within a standard sample block. Comparing whole slide counts of step sections from a given tumor block, CD8+ T cell percentage varied within 8% between all levels of a given breast cancer sample and within 2% between all levels of a given colorectal cancer sample. Also, we find, based on a linear-effects model and variance component analysis, that of any observed variability across all samples or within a tumor type, less than 1% can be attributed to section-to-section variability. We conclude that, for this particular IHC assay, CD8+ cell prevalence on a single slide can be representative of the CD8+ status of the entire block.

While evaluation of CD8 prevalence on a standard slide may generate consistent results, the adequacy of biopsy samples for evaluation of immune cell prevalence has not been extensively characterized. Biopsy sampling, particularly core biopsy sampling, has traditionally been examined in the context of solid tumor diagnosis and phenotyping. Additionally, core biopsies are routinely employed for assessing changes in immune cell numbers and activity in immunotherapy studies. In breast cancer, core biopsies have been shown to correlate poorly with whole slide sections for assessment of some histologic markers such as Ki-67 but well with others such as ER and HER2 [52]. As well, standard colon biopsies have been shown to harbor neoplastic lesions in deeper block levels in a minority of cases [53] and core biopsy samples of metastatic colorectal cancer lesions have shown significant molecular heterogeneity from the parent lesion, consistent either with tumor evolution or sampling bias [54]. By random *in silico* designation and analysis of 2 mm^2^ sections of tumor area to simulate biopsy sampling, we found that (1) one biopsy sample can approximate CD8 prevalence to within one standard deviation of the block mean in greater than 75% of all analyzed cases, (2) using multiple cores improves performance up to three cores (best), and (3) the mean CD8+ cell percentage for multiple biopsies was a less variable and less biased predictor of CD8 status for a given tumor sample than the maximum value. For three of four cases in which a single simulated core biopsy failed to estimate the CD8 prevalence to within 1 standard deviation of the corresponding slide or block, a heterogeneous distribution of CD8+ cells, concentrated primarily at the tumor margins, was noted in each slide of each case. Significant tissue loss was noted in one remaining case. This suggests that spatial heterogeneity can potentially confound interpretation of biopsies for immune cell prevalence but only in a minority of cases. However, we find that additional biopsy samples can significantly improve accuracy. For instance, where single core simulations in one case generated estimates to within 1 SD in only approximately 65% of instances, addition of a second core and calculation of mean CD8+ cell percentage generated accuracy to within 1 SD of the slide mean in approximately 85% of instances. Addition of a third core further improved accuracy to over 90%. Such improvements were observed in all cases where single biopsy simulations were accurate to within 1 SD less than 80% of the time.

However, if multiple biopsies are available for interpretation, is calculation of a mean CD8+ cell percentage the best for interpretation? We explored the utility of taking the maximum CD8+ cell percentage value obtained from multiple cores but found calculation of mean CD8+ cell percentage to be more accurate. With increasing numbers of samples, the mean CD8+ cell percentage reduces variability over the dynamic range of the assay and improves sensitivity with minimal change in a low diagnostic false positive rate across multiple diagnostic cut-offs (1%, 2%, 5%, 10%). As such, while mean CD8+ cell percentage is a highly specific method, sensitivity can be reduced at higher diagnostic cutoffs. Conversely, when using maximum CD8+ cell percentage, increasing the number of samples analyzed improves sensitivity but dramatically reduces specificity across all cut-offs. These relationships hold true for biopsy sample estimates relative to both corresponding slides as well as blocks.

Since samples for the current study were procured, the availability of tumor sections of adequate amount and integrity was limited and only less than 15 cases for each tumor type could be evaluated. Expanding the number of samples could better characterize any potential variability in step sections or biopsy samples. However, given the high degree of consistency in the existing samples, we speculate that any further differences would be minor and that the limited number of samples in the current study does not significantly detract from the results. Also, in the current study we could only examine one block from a given tumor. While the degree of cytotoxic T cell heterogeneity was not significant within a given block, we cannot make conclusions regarding the homogeneity of CD8 infiltration within an entire tumor. At the time of this writing, the degree of histologic CD8 heterogeneity from standard pathology sampling of an entire tumor specimen has not been documented. Performing the current analysis across multiple blocks/sections from a tumor would add further insight into the prognostic value of CD8 immunohistochemistry in tumors as well as potentially inform sampling methods by pathology services for routine histologic analysis. In addition, our methods may be more widely applicable and inform development of other tissue-based biomarkers. For instance, Foxp3+ cell prevalence in tumor samples either alone or in ratio to CD8+ cell counts has gained attention as a potential tissue-based prognostic marker in multiple tumor types. Studies in breast cancer have documented improved pathologic complete remission (pCR) and disease-free survival in HER2+ or triple negative breast cancer (TNBC) patients with CD8+ to Foxp3+ cell ratio (CFR) greater than 1 when assessed by immunohistochemistry [55]. As well, improved recurrence-free survival and breast cancer specific survival in TNBC patients without pCR has been documented [56]. For colon cancer, prognostic value of the CFR has been variable with some studies citing improved overall (OS) (Suzuki) and/or disease-free (DFS) survivals [57, 58] but others finding no association between the CFR and outcome [59]. In addition, immunohistochemical studies examining the prognostic significance of Foxp3+ cell [prevalence alone have been similarly variable finding either no association with outcome [58] or improved outcome for increased numbers of Tregs in tumor tissue and impaired outcome for increased numbers of Tregs in normal mucosa [60]. The variability of these results is attributable to multiple factors including different patient populations, biopsy type, disease stages, treatments, and metastatic status. The utility of Foxp3 as a Treg-specific marker has also been questioned since it has been shown to be expressed in non-suppressive activated T cells [61]. However, studies have not currently ruled out the possibility that spatial heterogeneity of FoxP3+ cells in tumor samples may also be a contributing factor. Histologic and digital image analyses similar to those we describe could clarify these findings and inform use of the CFR as a prognostic marker.

Despite the limitations associated with the number of tumor samples included in this analysis, our results highlight the practical importance of understanding *a priori* the analytical performance of any biomarker assay that may be deployed in clinical practice. An assessment of assay performance can also include the effect of biopsy type (resections, slides or core biopsies) on the results of the assay. Additionally, information about assay performance can also be included or accounted for when designing a clinical trial. While variability over the dynamic range of the assay in a patient population may be informative, it is of particular interest to understand assay performance around a given cut-off. Simulations can be run in order to assess if the assumed biomarker-treatment effect relationship of a therapy being interrogated is robust to assay performance issues given the cut-off and the range of expression in the patient population of interest. Once the clinical trial is ongoing, (blinded) assay data may also be evaluated in order to ensure that biomarker prevalence estimates and assay performance is as expected as during the design stages of the clinical trial and to guide potential risk mitigation strategies during enrollment.

## Conclusions

Taken together, our findings show that CD8+ cell prevalence is highly homogeneous within a standard tissue sample and that evaluation of any slide from a given block can be considered representative of the CD8+ cell prevalence. Also, through *in silico* biopsy modeling, we find that immune cell prevalence estimates can be accurately represented with just one biopsy sample and that accuracy improves with increasing sample number when the mean CD8+ cell percentage among the biopsy samples is assessed. These findings not only support the technical validity of biomarker strategies relying on CD8 immunohistochemistry but also suggest standards for sample number adequacy and interpretation.

## Supporting information

S1 Fig CD8 cores by slide. CD8 IHC repeated measures staining results for simulated core needles biopsies on 183 slides sectioned from 25 breast (red, left) and colorectal (blue, right) carcinoma samples.
(EPS)

S2 Fig CD8 cores by patient. CD8 IHC repeated measures staining results for simulated core needles biopsies for 25 breast (red) and colorectal (blue) carcinoma samples.
(EPS)

S3 Fig CD8 sampling and classification. Violin plots of overall percent agreement between CD8-positive and CD8-negative calls at the tumor (block) level when (i) using the original set of samples and resampling sections only (top), and (ii) when resampling both patients and then sections within patient (bottom). Scenario (i) captures technical reproducibility in this data set, while scenario (ii) attempts to capture variability in agreement calls in future samples of the same size. Treating the block-level mean as the gold standard "true" value of CD8 expression for that sample, using even just one slide (as opposed to averaging or taking the max of two slides) resulted in high agreement when applying a median (50th percentile) cut-off. Red points indicate a subsample of results over rounds of simulation.
(EPS)

## Abbreviations

ANOVA: analysis of variance
FFPE: formalin-fixed, paraffin-embedded
FPR: false positive rate
ICC: intra-class correlation coefficient
ROC: receiver operator characteristic
ROI: region of interest
SD: standard deviation
TIL(s): tumor infiltrating lymphocyte(s)
TPR: true positive rate
UICC: Union for International Cancer Control
